# Neuroinflammation aggravated by traumatic brain injury at high altitude is reversed by L-serine *via* NFAT1-mediated microglial polarization

**DOI:** 10.3389/fncel.2023.1152392

**Published:** 2023-04-14

**Authors:** Jinchun Liu, Shunhua Peng, Lisha Ye, Yechao Sun, Qiong Zhao, Hua Wei, Qianqian Luo, Min He, Guohua Wang

**Affiliations:** ^1^Department of Medicine, Henan Vocational College of Nursing, Anyang, Henan, China; ^2^Department of Physiology and Hypoxic Biomedicine, Institute of Special Environmental Medicine, Nantong University, Nantong, Jiangsu, China; ^3^Department of Basic Medicine, Yiyang Medical College, Yiyang, Hunan, China; ^4^Department of Scientific Research, Henan Vocational College of Nursing, Anyang, Henan, China

**Keywords:** traumatic brain injury, high altitude, L-serine, neuroprotection, microglia, nuclear factor of activated T-cell 1

## Abstract

Traumatic brain injury (TBI) is one of the main causes of disability and death, especially in plateau areas, where the degree of injury is often more serious than in plain areas. It is likely that high altitude (HA) aggravates neuroinflammation; however, prior studies are limited. This study was designed to evaluate the effects of HA on the degree of TBI and the neuroprotective effects and underlying mechanisms of L-serine against TBI at HA (HA-TBI). *In in vivo* experiments, wild-type mice and mice with *Nfat1* (*Nfat1^−/−^*) deficiency in the C57BL/6 background were kept in a hypobaric chamber for 3 days under simulated conditions of 4,000 m, 6,000 m and 8,000 m above sea level. After leaving the chamber, the standardized TBI model was established immediately. Mice were then intraperitoneally injected with L-serine (342 mg.kg^−1^) 2 h after TBI and then daily for 5 days. Behavioral tests and histological analysis were assessed at different time points post TBI induction. *In vitro*, we applied primary cultured microglia for hypoxia treatment (1% O_2_ for 24 h). The major findings include the following: (1) with increasing altitude, the neurological function of TBI mice decreased, and the damage to cerebral gray matter and white matter became more significant, (2) L-serine significantly improved the sensorimotor function of mice, reversed the increase in brain lesion volume, and promoted the renovation of brain tissue after HA-TBI, (3) L-serine significantly decreased the activation of microglia and promoted microglia polarization toward the protective M2 phenotype both *in vivo* and *in vitro*, (4) L-serine significantly suppressed the expression of NFAT1 in mice after HA-TBI and inhibited NFAT1 expression in primary microglia after hypoxia, and (5) knockout of *Nfat1* inhibited the inflammatory reaction caused by excessive activation of microglia, and L-serine lost its neuroprotective effect in *Nfat1* knockout mice. The present study suggests that HA aggravates brain damage after TBI and that the damage also increases with increasing altitude. As an endogenous amino acid, L-serine may be a neuroprotective agent against HA-TBI, and suppression of NFAT1 in microglia is a potential therapy for neuroinflammation in the future.

## 1. Introduction

Traumatic brain injury (TBI) has brought a huge economic burden to society, as well as physical and mental burdens to victims and their caregivers (Wang et al., 2013; Maas et al., 2022). TBI at high altitude (HA) refers to TBI that occurs in areas above 3,000 m above sea level (HA-TBI) (Yang et al., 2022). The increase in the number of people travelling to HA for work or adventure tourism makes HA-TBI of greater concern (Yu et al., 2014). The WHO estimates that 3.5 million individuals a year travel to areas over 3,000 m in elevation (Clarke, 2006). Because of the special environment of barometric pressure, which causes a reduction in the oxygen partial pressure, HA-TBI is followed by inflammation, apoptosis, necrosis, erythrocyte lysis and brain edema (Ahmad et al., 2013; Wang et al., 2021), all of which result in more severe clinical outcomes after HA-TBI (Lozano et al., 2015).

Among many great changes, such as the reduction of temperature and ambient humidity, the environmental characteristic defined by HA is the reduction of atmospheric pressure, which leads to the reduction of oxygen partial pressure at each point of the oxygen transport cascade from ambient air to cell mitochondria. People under HA may experience three forms of acute altitude illness: acute mountain sickness, high-altitude cerebral edema and high-altitude pulmonary edema (Luks et al., 2017), which may increase mortality and aggravate any kind of injury, including TBI. The basic pathological changes after TBI include primary injury and secondary injury (Simon et al., 2017). The mechanisms of secondary injury include inflammatory reactions, apoptosis, necrosis, erythrolysis, and the formation of brain edema (Hammad et al., 2018). Inflammation plays an important role in secondary injury following TBI (Simon et al., 2017; Hammad et al., 2018). The inflammatory mechanism mainly involves microglial activation, infiltration of inflammatory cells, and release of chemokines and cytokines (Corrigan et al., 2016). In the condition of HA, hypoxia can further induce secondary inflammation and aggravate the injury of the nervous system, leading to injury of gray and white matter, the destruction of neurobehavioral function, and cognitive impairment (Wang et al., 2019).

Microglia are crucial effector cells related to the immune response in the central nervous system (CNS) and play important roles in the inflammatory response (Corrigan et al., 2016). During early injury, microglia release a variety of substances, such as glutamate transporters, antioxidants and anti-inflammatory factors (Corrigan et al., 2016). They migrate to the damaged site to “rescue” slightly damaged cells or “destroy” severely damaged cells, playing a neuroprotective role through phagocytosis of cell debris and clearance of toxic substances (Xue and Yong, 2020). However, overactivation of microglia changes their morphology and function (proliferation, migration and phagocytosis), upregulates the expression of proinflammatory proteins, and produces a large number of reactive oxygen species, such as nitric oxide and tumor necrosis factor α, resulting in severe inflammatory reactions (Hu et al., 2015). Microglia/macrophages mainly include M1 and M2 polarized phenotypes (Loane and Byrnes, 2010). The proinflammatory M1 phenotype is conducive to the production and release of cytokines, thus aggravating nerve injury (Boche et al., 2013). Conversely, the M2 phenotype promotes the release of neurotrophic factors and functional recovery (Boche et al., 2013). We have previously found that the response of microglia/macrophages to TBI is a transient M2 phenotype, followed by a shift to M1, which is correlated positively with the severity of TBI (Wang et al., 2013, 2015). There have been many studies on the regulation of M1 and M2 polarization after brain injury. However, studies on the regulation of microglial polarization after HA-TBI have seldom been reported. The treatment of priming microglia/macrophages toward the beneficial M2 phenotype may provide a new anti-inflammatory strategy against HA-TBI.

Nuclear factors of activated T cells (NFATs) belong to a family of transcription factors and are usually composed of five members: NFAT1-5. In addition to NFAT5, NFAT1-4 act as general intracellular calcium sensors and transmit extracellular stimuli to the gene expression mechanism (Mognol et al., 2016). NFAT subtypes are expressed in various immune cells and guide the proliferation of different immune cells and tumor cells (Saika et al., 2020). When the cell is in the normal state, NFAT is in a highly phosphorylated state due to the action of the intracellular kinases CK1 and DYRK2 (Putney, 2012). Calcineurin is a serine/threonine calcium-dependent phosphatase that dephosphorylates and induces the nuclear translocation of NFAT1 (Jiang et al., 2022). When the nervous system is damaged, due to intracellular calcium overload, calcineurin dephosphorylates NFAT to induce its nuclear translocation and transcriptional regulation (Kurauchi et al., 2017; Jiang et al., 2022), including activation of HIF1α (Goyal et al., 2012). Hypobaric hypoxia at high altitude triggers the expression of HIF1α and inflammatory mediators (Goyal et al., 2012; Ahmad et al., 2013). NFAT1 is the major regulator of immune cell proliferation and microglial differentiation (Jiang et al., 2022). Whether NFAT1 is an upstream regulator in the pathogenesis of HA-TBI and whether NFAT1 regulates the microglial inflammatory (polarization) response after HA-TBI are worth studying.

As an important neurotrophic factor, L-serine can not only promote the proliferation and differentiation of neural stem cells (NSCs) but also play important roles in maintaining cell function and cell proliferation (Ye et al., 2021). It has been reported that L-serine induces M1 microglia to differentiate into the M2 phenotype in the early stage of injury, thus inhibiting the release of inflammatory factors to relieve excessive inflammatory reactions (Ding et al., 2020; Ye et al., 2021). In the present study, we found that the damage degree of HA-TBI was higher than that of plain TBI, and the total damage also increased with increasing altitude. In terms of the pharmacodynamics of L-serine, 6,000 m HA-TBI was selected as the research height to estimate the treatment effects of L-serine, and we found that L-serine serves as a neuroprotective agent for HA-TBI by modulating the polarization of microglia, thus inhibiting the cascade effect of inflammatory mediators after HA-TBI. We preliminarily speculated that L-serine might mediate the polarization of microglia by downregulating NFAT1 and suggested a potential therapeutic strategy against HA-TBI.

## 2. Materials and methods

### 2.1. Experimental animals

Male C57BL/6 mice weighing 25–30 g (8–10 weeks) and SD pregnant rats (18 days) were supported by the Animal Center of Nantong University. *Nfat1^−/−^* mice supplied by Cyagen Biosciences Inc. were a gift from Professor Yong-Jing Gao at Nantong University. *Nfat1^−/−^* mice were mated, and DNA was extracted from the newborn mice for genotype identification. The mice were well fed and housed in a pathogen-free and climate-controlled environment at room temperature under a 12-h light–dark cycle. All experiments were performed in line with the Guidelines for the Care and Use of Experimental Animals of the National Institutes of Health and approved by the Animal Ethics Committee of Nantong University (S20190316-021).

### 2.2. Preparation of high-altitude (HA) TBI and grouping

**Figure 1:**
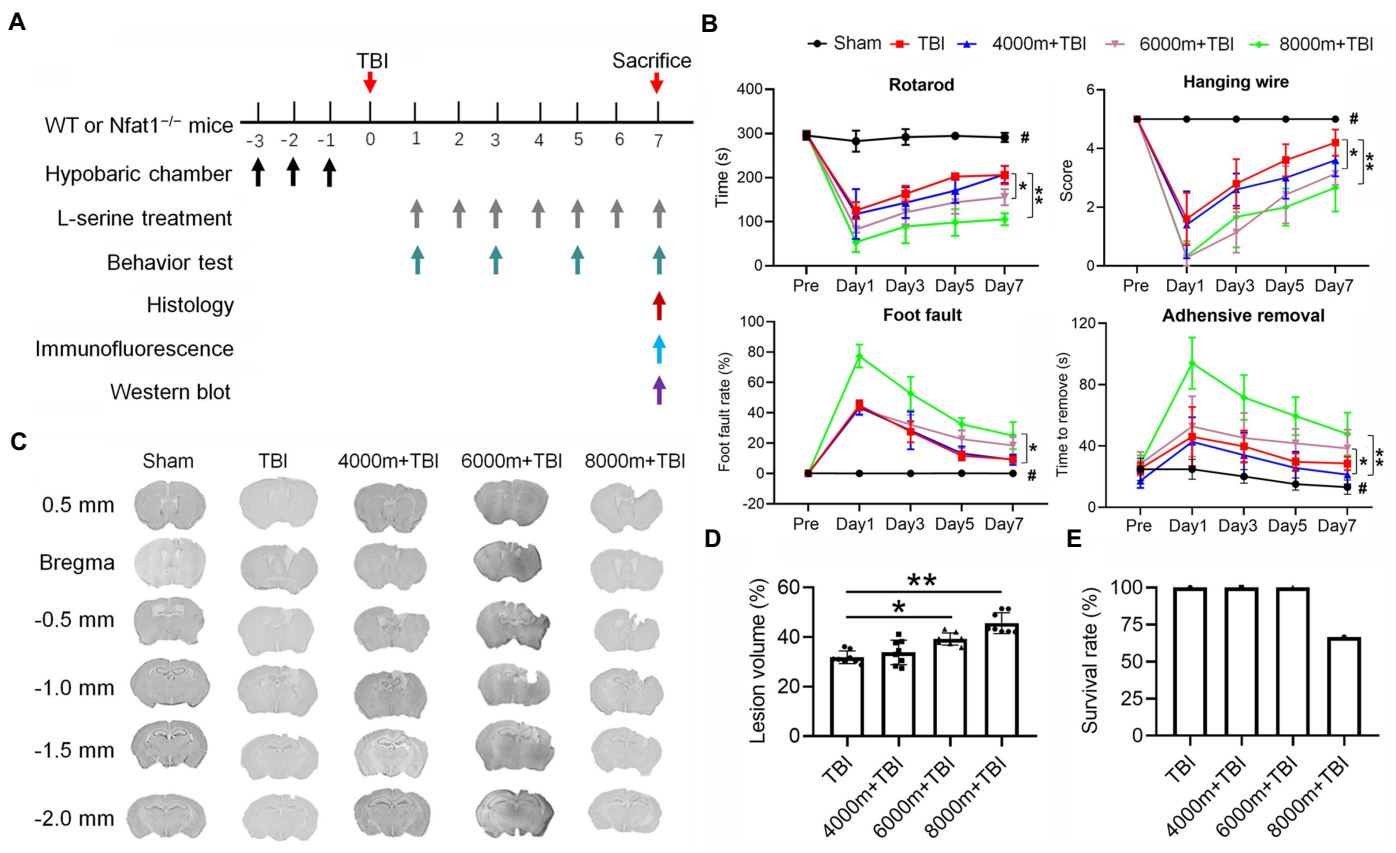
Effects of different altitudes on neurobehavioral function, cerebral gray matter and cerebral white matter in mice following TBI. **(A)** Temporal schematic of the experimental protocol and the observation time points. **(B)** Effects of different altitudes on neurobehavioral function in mice (*n* = 7–8). **(C)** Coronal section of brain slices from bregma 0.5 mm to −2.0 mm, showing the lesion brain tissue at different altitudes. **(D)** Quantification of the lesion volume (*n* = 8). **(E)** Effect of TBI at different altitudes on the survival rate of mice (*n* = 8). ^*^p < 0.05, ^**^p < 0.01 versus the indicated group; ^#^*p* < 0.01 versus other groups.

In this study, simulation conditions at different high altitudes (HAs) and HA-TBI (TBI at HA) models were first established. The mice were randomly divided into the sham-operated group at sea level (sham group), TBI at sea level group (TBI group), 4,000 m TBI group (4,000 m + TBI), 6,000 m TBI group (6,000 m + TBI), and 8,000 m TBI group (8,000 m + TBI). Mice were first placed into the decompression chamber (Shanghai Tawang Intelligent Technology Co., Ltd., China) 3 days before the operation. The oxygen concentration was 24–25%, the temperature was 20–24°C, and the humidity was 50–55%. Different oxygen partial pressures were selected to simulate the 4,000 m altitude, 6,000 m altitude, and 8,000 m altitude environments. In detail, the oxygen partial pressure was 12.91 kPa at 4,000 m, 9.72 kPa at 6,000 m, and 7.51 kPa at 8,000 m. After 3 days of HA exposure, the mice were removed from the chamber and anaesthetized with 1.5% isoflurane in a 30% O_2_/68.5% N_2_O mixture. HA-TBI was then induced using the controlled cortical impact (CCI) procedure, which was carried out as described in our previous works (Wang et al., 2013, 2015). The animals were fixed on the brain stereotactic instrument, and the skull was opened with a 3-mm diameter incision. The center of the incision was located 1.5 mm lateral to the midline and 0.5 mm anterior to bregma. A 3-mm flat-tip impounder was dropped onto the mouse incision with a contact time of 150 ms, an impact speed of 3.5 m/s and an impact depth of 1.5 mm by using a pneumatically driven CCI device (Precision Systems and Instrumentation, Fairfax, VA, United States). Immediately after CCI, the mouse bone flap was replaced, and the scalp was sutured. When the mice were about to wake up, they were sent back to the decompression chamber for another day and then returned to normal feeding. At a specific time point (Figure 1A), neurobehavioral assessments and histological experiments were performed to evaluate the severity of TBI.

Based on the above results, we chose a simulated HA environment at an altitude of 6,000 m to study the neuroprotective effects and mechanisms of L-serine in the following experiments. Mice were randomly divided into a sham group, a sham group at an altitude of 6,000 m (HA group), a 6,000 m TBI group (HA-TBI group) and a 6,000 m TBI + L-serine group (HA-TBI + L-ser group). As described previously (Ye et al., 2021), L-serine (Saint Louis, United States) was intraperitoneally injected at 342 mg/kg at 2 h after TBI and then twice each day for 7 days. Mice in other groups were treated with the same amount of saline.

### 2.3. Sensorimotor function assessments

All sensorimotor function tests were carried out by researchers who were blinded to the allocation of the experimental groups.

#### 2.3.1. Rotarod test

Adaptability training was carried out 3 days before modelling, and the set procedure was to accelerate from 4 rpm to 40 rpm in 300 s twice a day until the mouse did not fall off the shaft. Tests started 1, 3, 5, and 7 days after modelling. The roller accelerated from 4 rpm to 40 rpm in 300 s. The latency time was recorded as the time when each mouse fell from the shaft or hung passively on the shaft to rotate with the shaft. Each test was repeated three times.

#### 2.3.2. Hanging wire test

The hanging wire test of the device is a stainless-steel crossbar with a length of 50 cm and a diameter of 2 mm, which is placed on two vertical supports 37 cm above the plane. The mouse was placed on the crossbar and observed for 30 s, and the amount of time spent hanging was recorded (repeated four times). According to the performance of each mouse, the score is 0–4 points: 0, mouse falls off the crossbar; 1, mouse hangs on the bar with two forepaws; 2, mouse hangs on the bar and tries to climb up the bar; 3, mouse not only uses two forepaws but also uses one or two rear claws to hang on the bar; 4, mouse hangs on the bar, with forepaws and tail wrapped around the bar; and 5, mouse can escape to any bracket.

#### 2.3.3. Foot fault test

The foot fault test was used to assess deficits in forelimb and hindlimb function. The mouse was placed on the enhanced grid surface (30 l × 35 W × 31 H cm) with a 2.5 cm^2^ grid opening. Each fall and slide between the steel wires with weight-bearing steps was recorded as foot failure of the hindlimb or forelimb. Foot fault rate = number of wrong steps/total number of steps × 100%.

#### 2.3.4. Adhesive test

Each animal was trained to tear off the adhesive strip for 3 days. In the training of removing sticky strips, sticky labels with a length of approximately 1 cm and width of approximately 1 cm were used to stick to the right forelimb of the animal. The animal was taken out of the cage, and the label was used on its right limb. Once the label was attached to the limb, the animals were placed in a clean experimental cage. During training, the animals used their mouths and moved another forelimb in a small range to remove the label. During the test, the time when the mouse first started rubbing its fingers was recorded as the feeling time, and the time when the mouse finally rubbed the sticky strip down was recorded as the movement time.

### 2.4. Measurements of TBI volume

A Leica camera (DM5000B, Leica, Bensheim, Germany) was used to collect the photographs of brain slices for measuring the volume of lesions using ImageJ analysis software (National Institutes of Health, United States). The volume percentage of lesions was calculated using the formula ((VC − VL)/VC) × 100% by a researcher who was blinded to the experimental groups; VL = volume of uninjured tissue in the right hemisphere subjected to TBI, while VC = volume of the left hemisphere (uninjured side).

### 2.5. Luxol fast blue staining

As previously described (Wang et al., 2019), the postfixed brains were dried and protected in 30% sucrose in PBS. According to the manufacturer’s instructions, coronal sections (25 μm thickness) were stained with Luxol fast blue (American Mastertech, Lodi, CA, United States) to detect myelin injury. Briefly, slides were incubated in a prewarmed Luxol fast blue (60°C) solution for 1 h. After washing with water, slides were differentiated in lithium carbonate solution for 30 s and then with 70% ethanol for 30 s. The slides were finally sealed with coverslips for microscopic examination.

### 2.6. Nissl staining

Nissl staining was performed with a kit according to the manufacturer’s instructions (Beyotime, Cat# C0117, Shanghai, China). Briefly, sections (bregma 0.5 mm to −2.0 mm) were placed in cresyl violet solution at 37°C for 8 min, washed with distilled water twice for 30 s, dehydrated twice with 95 and 100% ethanol for 2 min, cleared three times with xylene for 3 min, and sealed with neutral gum for microscopic examination.

### 2.7. PCR

The *Nfat1* gene (NCBI Reference Sequence: NM_010899.3; Ensembl: ENSMUSG00000027544) is located on chromosome 2 in mice (Jiang et al., 2022). Ear DNA was extracted with the phenol–chloroform method to identify WT or *Nfat1* knockout mice. DNA (≈500 ng) was amplified by a Taq PCR MasterMix (Vazyme Biotech Co., Nanjing, China) and 1 μM primers. The sequences of the primers were as follows: forward CAGACTCAGGGCACCAAGAGGAAA; reverse GGGTGCCCTTGTCCATAATAGTAG. The extracted DNA and primers were first denatured at 94°C for 4 min, followed by 25 cycles at 94°C for 30 s, 60°C for 30 s, 72°C for 50 s, and finally extended at 72°C for 5 min. The amplicons were isolated with 1.5% agarose gel, stained with GelRed and photographed by the GelDoc-It imaging system (UVP Co., Ltd., Upland, CA, United States).

The total RNA of brain tissue was extracted using TRIzol reagent (Invitrogen, Carlsbad, CA, United States) for mRNA expression. Quantitative PCR analysis was carried out by AceQ qPCR SYBR Green Master Mix (Vazyme) in a real-time detection system. The primer sequences were as follows: CD16: TTTGGACACCCAGATGTTTCAG and GTCTTCCTTGAGCACCTGGATC; CD206: CAAGGAAGGTTGGCATTTGT and CCTTTCAGTCCTTTGCAAGC. PCR amplification was performed at 95°C for 3 min, followed by 40 thermal cycles at 95°C for 10 s and 60°C for 30 s. Melting curves were generated after completion of the cycles to ensure that there were no nonspecific products. Ct (cycle threshold) values were normalized to GAPDH Ct (control mRNA) and quantified using the 2^-ΔΔCT^ method.

### 2.8. Western blot

A BCA protein assay (SN209941, Bio-TEK, Durham, NC, United States) was used to determine the protein concentration of primary microglia by calculating the absorbance at 562 nm on a microplate reader (Bio-TEK). An extract containing approximately 30 μg of protein was equally divided onto a 10% separated SDS–PAGE and transmembrane using a Bio-Rad system. The blots were blocked in 5% nonfat milk and incubated with anti-NFAT-1 (1:1,000, Ab2722; Abcam, United States) and β-actin (1:10,000, A2228; Sigma–Aldrich, St. Louis, MO, United States) primary antibodies overnight at 4°C. After washing three times, the blots were incubated with goat anti-mouse IgG combined with horseradish peroxidase (HRP, diluted 1:1000; Pierce Biotechnology Inc., Rockford, IL) secondary antibody for 1 h at 37°C. The intensity of the specific bands was finally measured and analysed by an Odyssey infrared imaging system (Li-Cor Biosciences, Lincoln, NE, United States).

### 2.9. Immunofluorescent staining

According to a previous protocol (Wang et al., 2019), the sections were incubated with one of the following primary antibodies in blocking solution at 4°C overnight: rabbit antibody to myelin basic protein (MBP, 1:200, ab40390, Abcam); mouse anti-nonphosphorylated neurofilament protein (SMI-32, 1:200, NE1023, Millipore); rabbit anti-ionized anti-calcium binding adaptor molecule-1 protein (Iba-1, 1:5,000, 019–19,741, Wako); mouse anti-nuclear factor of activated T-cell 1 protein (NFAT1, 1:2,000, ab59256, Abcam); mouse anti-CD16 antibody (CD16, 1:200, BD Corporation); and mouse anti-CD206 antibody (CD 206, 1:200, R&D Corporation). The sections were subsequently incubated with secondary antibodies DAM-Alexa488 and DAR-Alexa555 at room temperature for 2 h. Fluorescence microscopy images were obtained with a computer-driven Leica microscope (DMLB, Leica Corporation, Solms, Germany) by Leica Qwin V3 software. The immunostaining intensity of MBP and SMI32 antibodies and the number of target immunopositive cells were quantified using ImageJ software. Three randomly selected microscopic fields in the CC area on each of three consecutive sections were analyzed for each mouse brain. White matter damage was quantified as the average ratio of SMI32 to MBP intensity.

### 2.10. Primary microglial cultures

Primary microglia were cultured according to a previous method (Wang et al., 2015). Briefly, microglia were isolated in shake flasks containing mixed glia at 180 g for 1 h and then collected and seeded at 3 × 10^6^/well in 6-well plates. Cultured primary microglia were washed twice with extracellular solution. The microglia were then incubated in serum-free DMEM at 37°C and maintained under either normoxic conditions (21% O_2_) or placed in an incubator (94% N_2_, 5% CO_2_ and 1% O_2_) for hypoxic exposure for 24 h as previously described (Wang X. et al., 2022). For *in vitro* L-serine treatment, different concentrations of L-serine (0, 10, 30, 100 and 300 μM) were added to the cultures for 3 days. Media were collected at selected times for measurements of NO production and TNF-α (see below). Protein and mRNA were harvested from cultured microglia for Western blot analysis and qRT–PCR.

### 2.11. Enzyme-linked immunosorbent assay (ELISA)

The protein levels of TNF-α were determined using ELISA kits (Cat. no. JP27194; Immuno-Biological Laboratories, Hamburg, Germany) based on the manufacturer’s protocol. Absorbance at 450 nm was detected within 30 min using an ELISA microplate reader (Bio-Tek).

### 2.12. Measurement of nitric oxide (No) levels

NO levels were calculated using an electrically isolated NO potentiometer (ISO-NO Mark II, World Precision Instruments, Inc., Sarasota, FL) and a 200 μM shielded sensor in which NO oxidizes the platinum-containing carbon fibre sensor to generate current. The ISO-NO system was calibrated daily with the NO donor SNAP (S-nitroso-n-acetyl-L-penicillamine) prior to each experiment. NO levels were calculated from the calibration curve of the generated current, expressed as the concentration change (nM) of the baseline value.

### 2.13. Statistical analyses

SPSS 23 software (IBM, Armonk, NY, United States) was applied for statistical analysis. The homogeneity of variance was checked before using a parametric test. All values were expressed as the mean ± standard deviation (SD). Significant differences between groups were assessed by one-way analysis of variance (ANOVA) and multiple comparisons of Tukey’s *post hoc* tests. Data from behavior tests were analyzed with two-way repeated-measures ANOVA and Tukey’s *post hoc* tests. *p* < 0.05 was considered statistically significant.

## 3. Results

### 3.1. Effects of different has on neurological function and lesion volume after TBI

After establishing TBI models at different altitudes, neurobehavioral functions were tested 1, 3, 5, and 7 days after modelling (Figure 1A). TBI elicited significant sensorimotor deficits in contrast with the sham group (Figure 1B, *p* < 0.01). Compared to the TBI group (Figure 1B), there were significant differences (*p* < 0.01 or *p* < 0.05) in the rotarod test, hanging wire test, adhesive test and foot fault test in the 6,000 m + TBI and 8,000 m + TBI group. However, there was no significant difference in neurobehavioral function between the 4,000 m + TBI group and TBI group (*p* ˃ 0.05). Consistently, the lesion volumes of the 6,000 m + TBI group and 8,000 m + TBI group were significantly aggravated compared with those of the TBI group (Figures 1C,D, *p* < 0.05). In the HA-TBI groups with an altitude of 4,000 m and 6,000 m, no mice died within 7 days after TBI, but in the HA-TBI group with an altitude of 8,000 m, the mortality rate was as high as 33% (Figure 1E).

### 3.2. Effects of different has on gray and white matter injury after TBI

**Figure 2:**
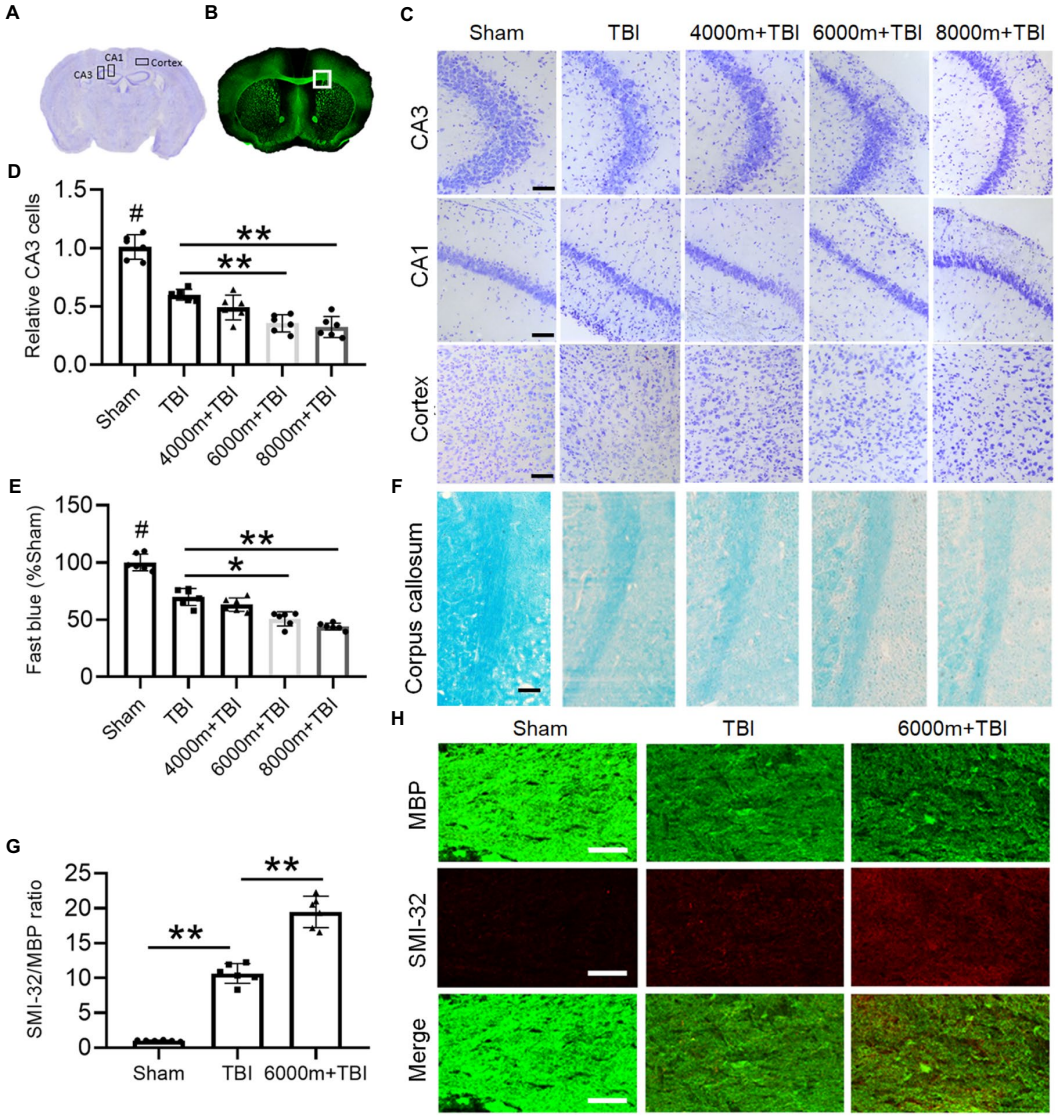
Effects of different altitudes on the degree of cerebral gray and white matter damage following TBI. **(A)** The regions of interest are CA3 and CA1 in the hippocampus and cortex of Nissl staining in a coronal section of the mouse brain. **(B)** The square region of interest is the corpus callosum (CC) area of MBP fluorescence staining in a coronal section of the mouse brain. **(C)** Nissl staining of the right side of CA3, CA1 and cortex in different groups, scale = 50 μm. **(D)** Relative quantification of viable CA3 cells (*n* = 6). **(E)** The integrated density of Luxol fast blue staining and normalized to sham animals (*n* = 6). **(F)** Representative fast blue staining of the CC, scale = 50 μm. **(G)** The degree of white matter injury was expressed as the ratio of SMI-32 to MBP (*n* = 6). **(H)** Double immunofluorescent staining for SMI-32 and MBP in the right CC, scale = 50 μm. ^*^p < 0.05, ^**^p < 0.01 versus the indicated group; ^#^*p* < 0.01 versus other groups.

We characterized the effects of HA exposure on TBI at the histological structure and cellular levels by Nissl (Figure 2A) and immunofluorescence staining (Figure 2B). Compared with the sham operation, the number of viable neurons was greatly reduced after TBI, as shown in the CA3 and CA1 areas of the hippocampus and in the ipsilateral cortex (Figure 2C). As expected, HA exposure significantly enhanced the damage of TBI with a marked decrease in CA3 neurons, especially at HAs of 6,000 m and 8,000 m (Figure 2D, *p* < 0.01). 6,000 and 8,000 m of HA exposure dramatically aggravated the TBI-induced reduction in the expression of myelin in the corpus callosum (CC), as shown by Luxol fast blue staining (Figures 2E,F, *p* < 0.05 or *p* < 0.01). Double immunofluorescence staining was also performed with anti-MBP and anti-SMI32 antibodies to further evaluate the pathology of myelin in the CC (Figure 2H). Compared with TBI alone, 6,000 m HA exposure increased SMI32 immunostaining and led to loss of MBP-positive myelin in mice, as shown by an increase in the SMI32/MBP ratio in the CC (Figure 2G, *p* < 0.01). These experiments strongly suggest that HA may aggravate TBI-induced necrosis of hippocampal neurons and myelin pathology.

### 3.3. Neuroprotective effects of L-serine treatment on HA-TBI in mice

**Figure 3:**
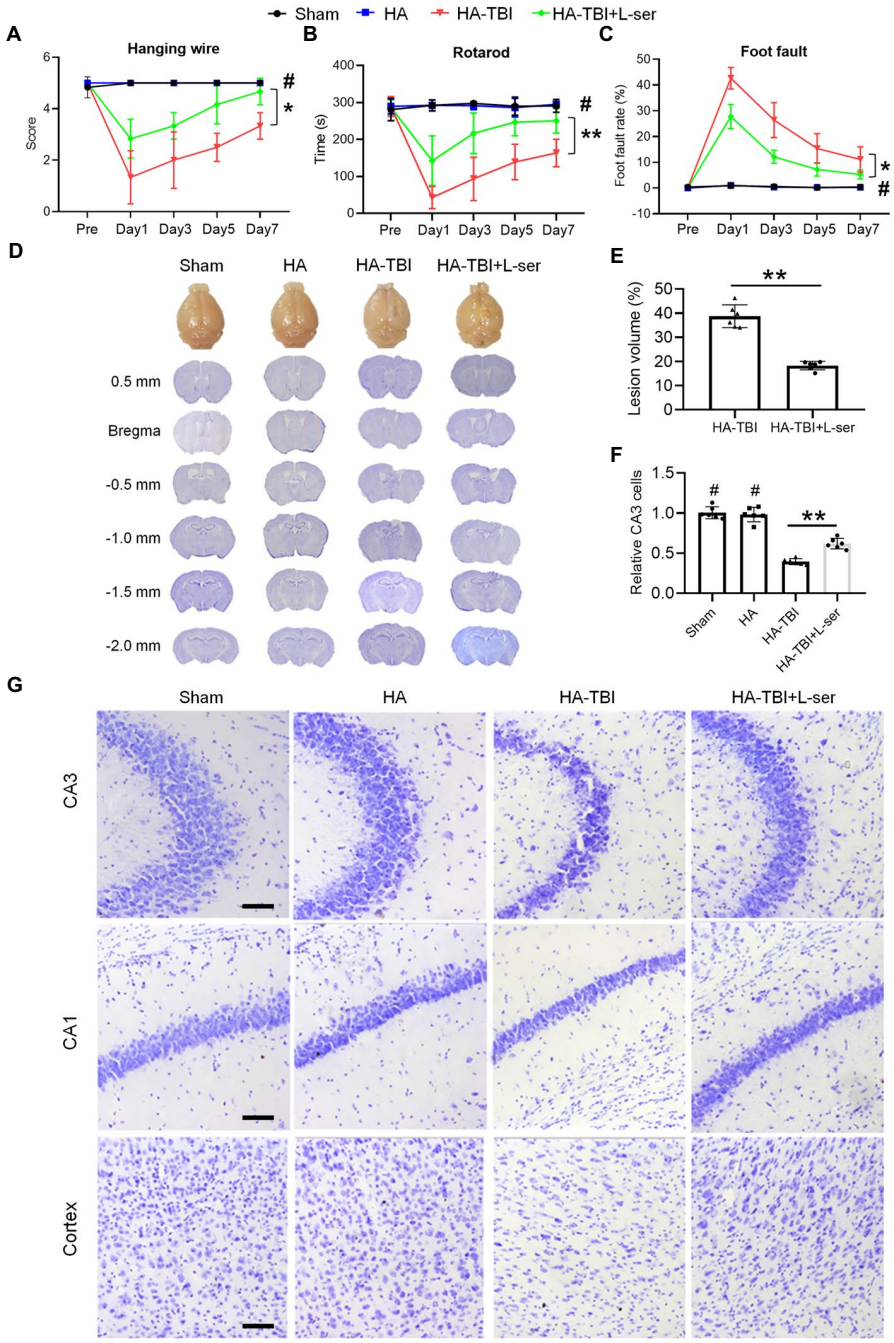
Effects of L-serine on neurobehavioral function in mice after HA-TBI and its neuroprotective effect on cerebral gray matter. **(A)** Hanging wire test (*n* = 7–8). **(B)** Rotarod test (*n* = 7–8). **(C)** Foot fault test (*n* = 7–8). **(D)** Nissl staining of coronal sections of brain slices from bregma 0.5 mm to −2.0 mm, showing the lesion of brain tissue in the different groups. **(E)** Quantification of the lesion volume (*n* = 6). **(F)** Relative quantification of viable CA3 cells (*n* = 6). **(G)** Representative Nissl staining of the right side of CA3, CA1 and cortex in different groups, scale = 50 μm. ^*^p < 0.05, ^**^p < 0.01 versus the indicated group; ^#^*p* < 0.01 versus other groups.

Based on the above results, we chose a simulated HA environment at an altitude of 6,000 m to study the pharmacology and mechanism of L-serine in the following experiments. Meanwhile, to eliminate the potential impact of HA on mice, we set up an additional altitude of 6,000 m (HA alone group). There was no significant difference in any detection index between the sham and HA groups (Figure 3, *p* > 0.05). Compared with the HA-TBI group, mice in the HA-TBI + L-ser group had significantly improved scores in the hanging wire test (Figure 3A, *p* < 0.05), increased the time spent on the rotating rod (Figure 3B, *p* < 0.01), and decreased the foot fault rate in the grid (Figure 3C, *p* < 0.05). In addition, L-serine treatment significantly decreased cortical lesion volume (Figures 3D,E, *p* < 0.01) and exhibited a notable number of viable neurons in hippocampal CA3 (Figures 3F,G, *p* < 0.01).

**Figure 4:**
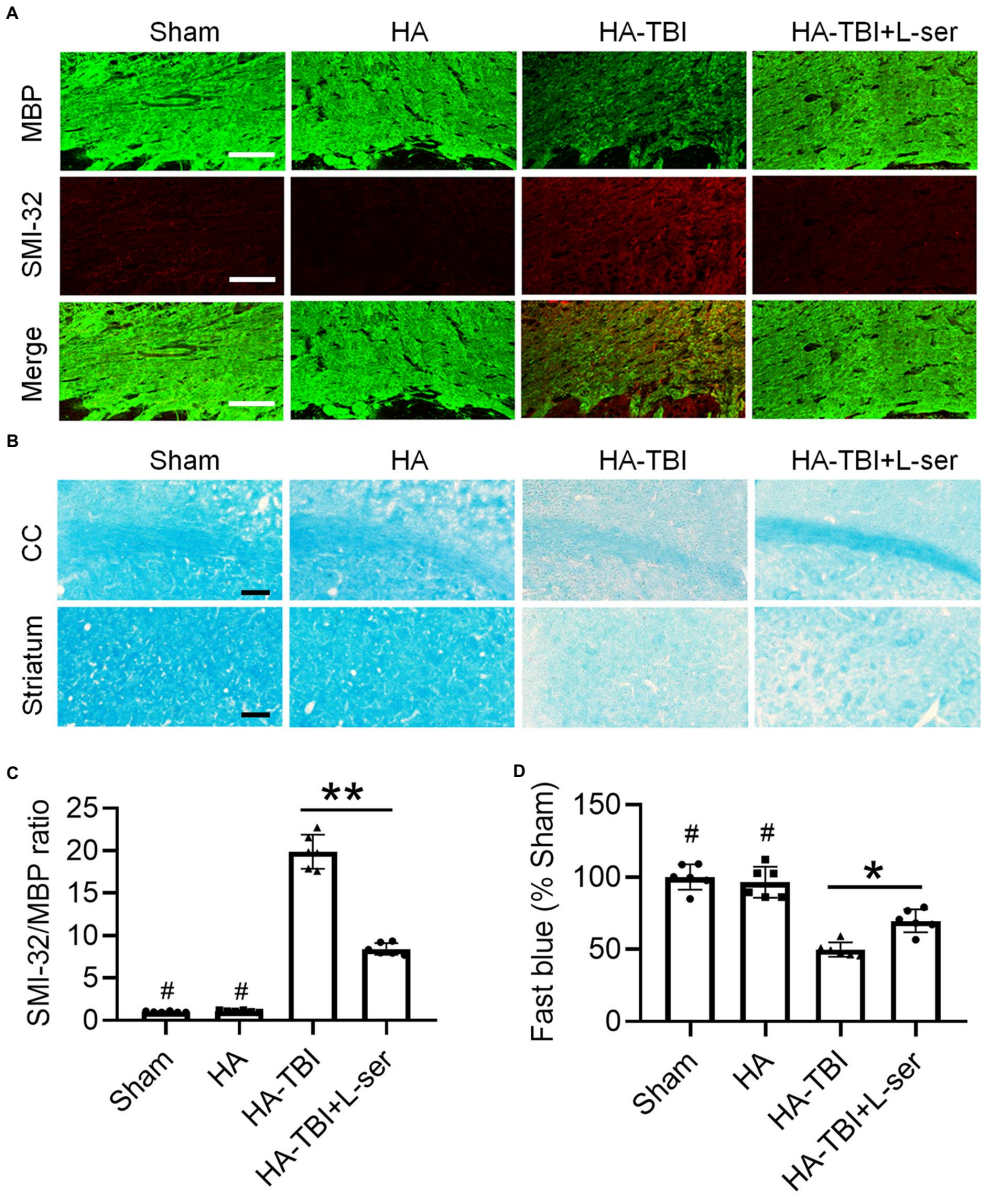
Effects of L-serine against white matter injury in mice after HA-TBI. **(A)** Double immunofluorescent staining for SMI-32 and MBP in the right CC in different groups. **(B)** Representative fast blue staining of the CC and striatum, scale = 50 μm. **(C)** Relative quantification of the ratio of SMI-32 to MBP (*n* = 6). **(D)** Quantification of Luxol fast blue staining normalized to sham animals (*n* = 6). ^*^p < 0.05, ^**^p < 0.01 versus the indicated group; ^#^*p* < 0.01 versus other groups.

Compared with the sham group and HA group, the MBP immunofluorescence staining intensity of the HA-TBI group was reduced, and SMI32 was increased at 7 days postinjury (Figures 4A,C). In contrast, L-serine-treated animals after HA-TBI showed an increase in myelin integrity and a decrease in the SMI32/MBP ratio in the CC (Figures 4A,C, *p* < 0.01). Similarly, the blue color of the CC and striatum in the HA-TBI group became lighter, indicating the loss of myelin sheath and demyelination; however, the blue fading of the CC and striatum was recovered in the L-ser treatment group (Figures 4B,D, *p* < 0.05).

### 3.4. Effects of L-serine on NFAT1 expression and microglial activation after HA-TBI *in vivo*

**Figure 5:**
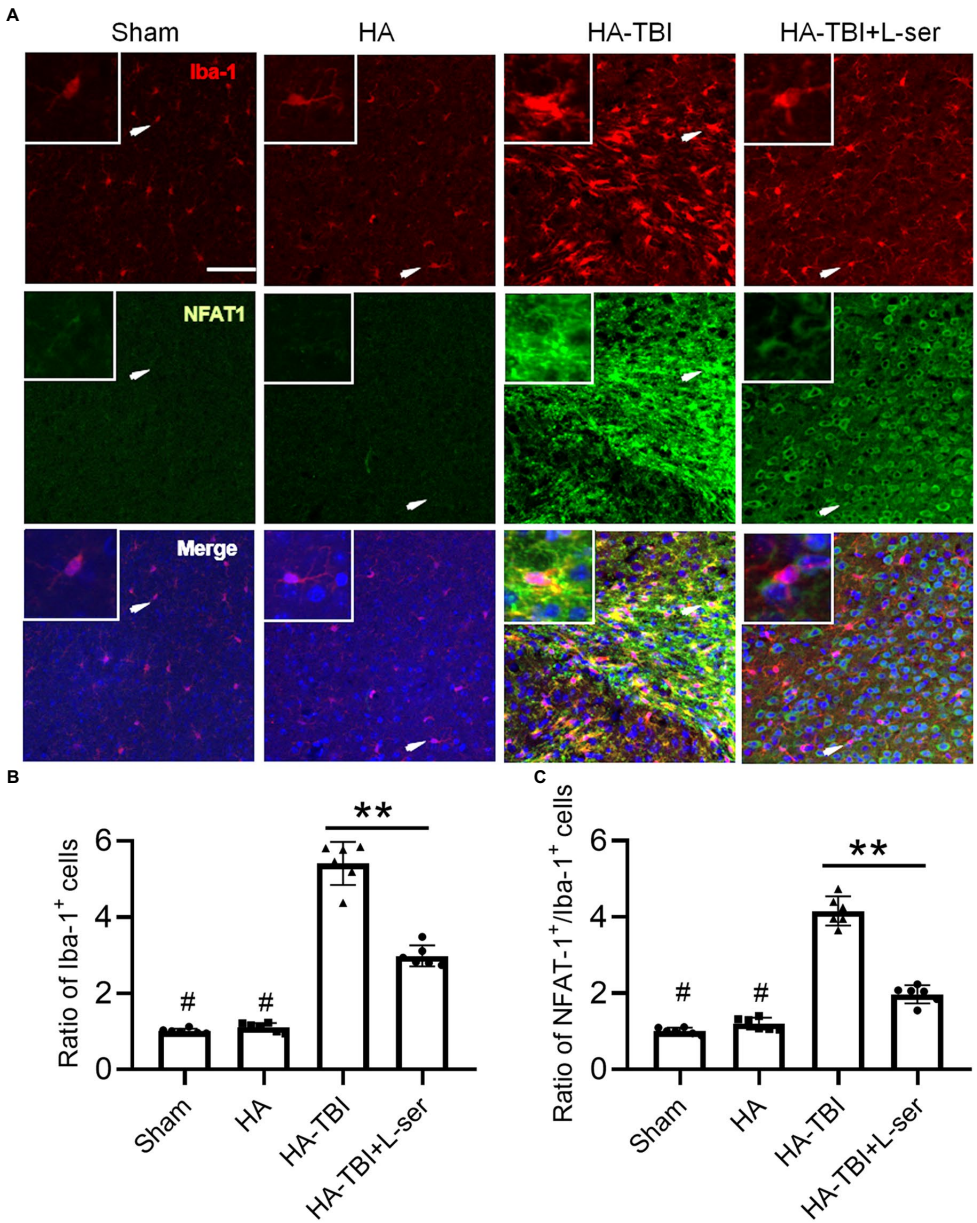
Effects of L-serine on the expression of Iba-1 and Nfat1 after HA-TBI. **(A)** Representative images of double immunofluorescence staining of NFAT1 with Iba-1 in the ipsilateral cortex in different groups. **(B)** Relative ratio of Iba-1-positive cells. **(C)** Relative counts of NFAT1+/Iba-1^+^ cells in the lesion sites. *n* = 6; scale = 50 μm. ^*^p < 0.05, ^**^p < 0.01 versus the indicated group; ^#^*p* < 0.01 versus other groups.

Compared with the sham group and HA group, the fluorescence intensity of NFAT1 and Iba-1 increased significantly in the HA-TBI group, but the effects were reversed significantly by treatment with L-serine (Figures 5A,B, *p* < 0.01). In terms of the morphology of microglia, the morphology of microglia in the HA-TBI group showed swelling of cell bodies and increased protrusions compared with the sham group and HA group. In addition, there were a large number of Iba-1-positive and activated NFAT1-positive microglia in the HA-TBI group, and L-serine significantly inhibited the activated microglia and suppressed the expression of NFAT1 (Figure 5C, *p* < 0.01).

### 3.5. Effects of L-serine on the inflammatory response of primary microglia in a hypoxic environment *in vitro*

**Figure 6:**
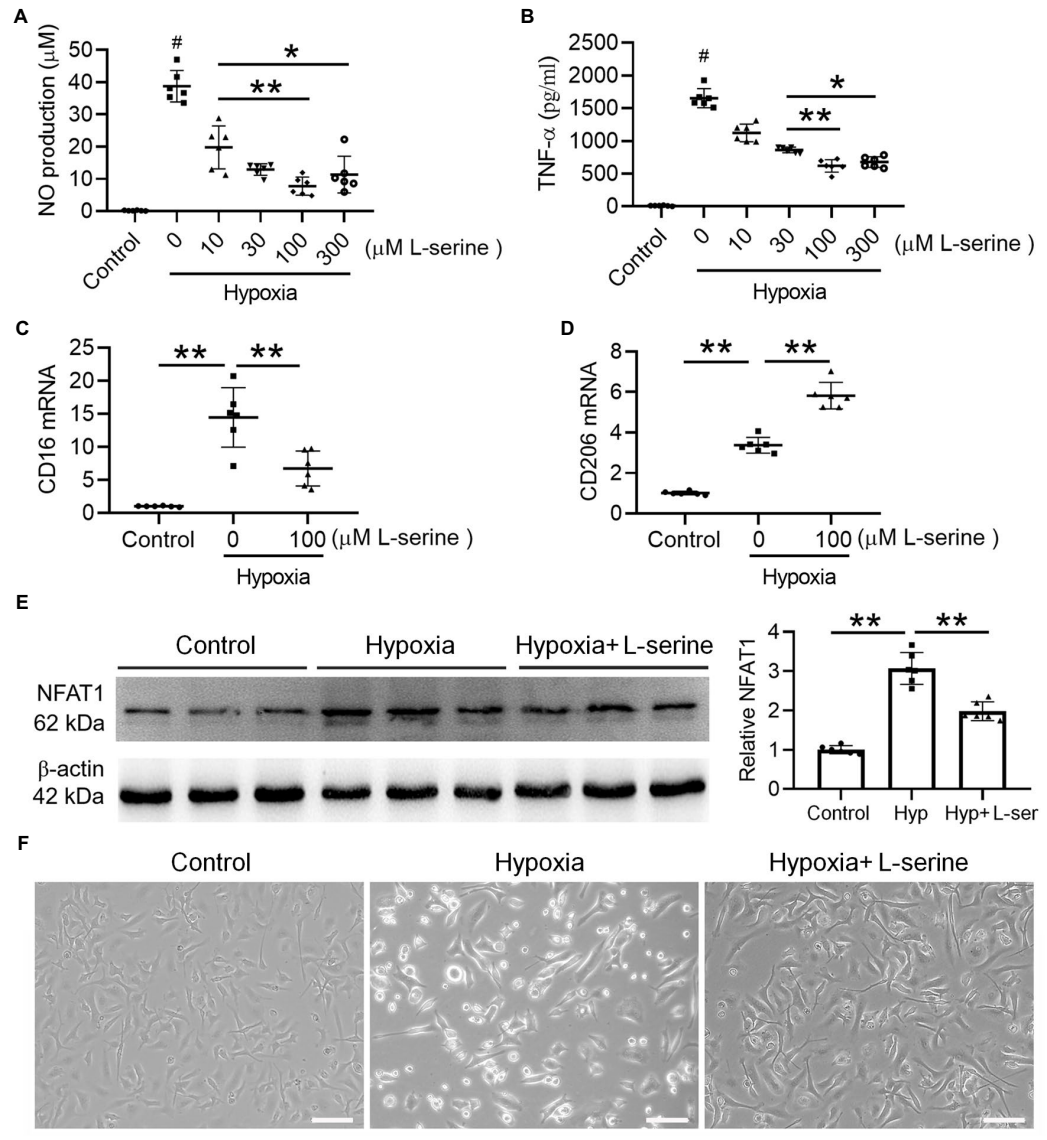
Effects of L-serine on inflammatory mediators, NFAT1 expression and microglial polarization after hypoxia treatment *in vitro*. **(A,B)** Levels of NO and TNF-α in LPS-treated microglia with or without different concentrations of L-serine. **(C,D)** Messenger RNA levels of CD16 and CD206 in cultured microglia after treatment with hypoxia *in vitro* with or without L-serine. **(E)** Protein expression levels of NFAT1 detected by Western blot in cultured microglia after treatment with hypoxia *in vitro* with or without L-serine. **(F)** Photomicrographs showing the morphology of mouse primary microglia in the three groups. *n* = 8–10. ^*^p < 0.05, ^**^p < 0.01 versus the indicated group; ^#^*p* < 0.01 versus other groups.

We further explored the underlying mechanism of hypoxia-induced microglial activation by exposure to 1% O_2_ for 24 h. As expected, the contents of NO (Figure 6A) and TNF-α (Figure 6B) increased significantly after hypoxia treatment, and the production of TNF-α and NO was attenuated by L-serine treatment in a dose-dependent manner (*p* < 0.01). Moreover, maximum anti-inflammatory effects from L-serine were observed at a concentration of 100 μM (Figures 6A,B). We further used 100 μM L-serine to investigate its effects on microglial polarization by qRT–PCR analysis (Figures 6C,D). As expected, the expression of CD16 (M1 type) mRNA increased significantly after treatment with hypoxia compared with the control group and was inhibited significantly by L-serine treatment (*p* < 0.01). In contrast, CD206 (M2 type) mRNA was significantly increased by L-serine treatment (*p* < 0.01), suggesting the role of L-serine in promoting microglial polarization toward the M2 type. Primary microglia became hypertrophic and vacuolated after hypoxia treatment, and L-serine partially recovered the morphological and pathological changes (Figure 6F), which was consistent with the above results. Compared with that in the control group, the expression of NFAT1 protein in the LPS group increased significantly but was inhibited by the addition of L-serine (Figure 6E, *p* < 0.01).

### 3.6. Effects of L-serine on the polarization of Nfat1 knockout mice after HA-TBI

**Figure 7:**
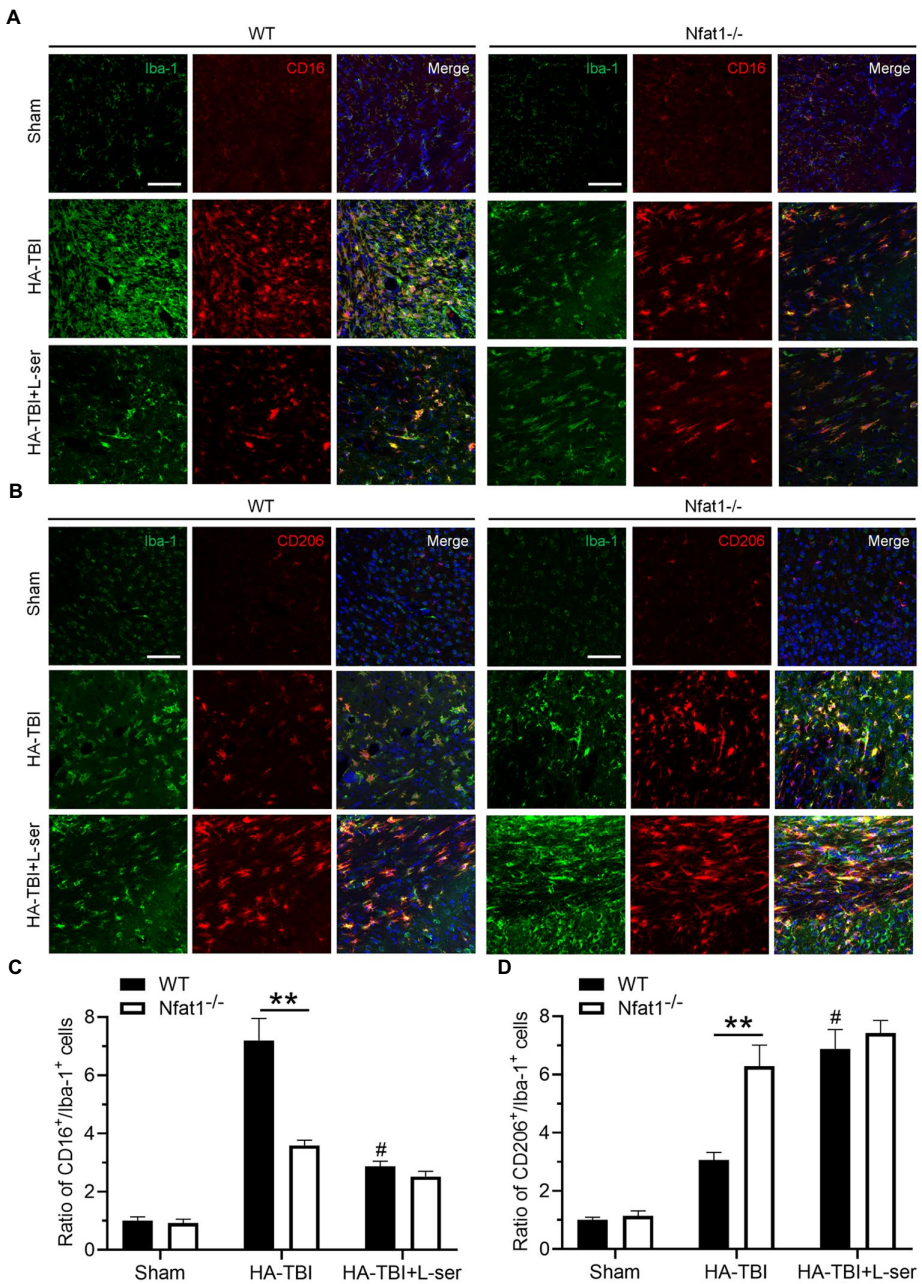
Effects of L-serine on the polarization of microglia in WT mice and *Nfat1* knockout mice after HA-TBI. **(A)** Representative images of double immunofluorescence staining of Iba-1 and CD16 in sections 0.5 mm anterior to the bregma in different groups 7 days post HA-TBI. **(B)** Representative images of double immunofluorescence staining of Iba-1 with CD206 at the section 0.5 mm anterior to the bregma in different groups 7 days post HA-TBI. **(C)** Relative cell counts of colabelled CD16^+^/Iba-1^+^ cells in the lesion sites shown in A. **(D)** Relative cell counts colabelled for CD206^+^/Iba-1^+^ in the lesion sites as shown in B. *n* = 6; Scale = 50 μm. ^**^p < 0.01 versus the indicated group; ^#^*p* < 0.01 versus the HA-TBI group of WT.

A 6,000 m TBI model was established in wild-type mice (WT group) and *Nfat1* knockout mice (*Nfat1*^−/−^ group). Similar to the results of C57BL/6 mice, HA-TBI increased the expression of Iba-1 in the WT group, but the activation of Iba-1 was inhibited by L-serine treatment (Figure 7, left). *Nfat1* deletion attenuated HA-TBI-induced sensorimotor function deficits and lesion volume (data not shown). Compared with that in WT mice, the number of activated microglia significantly decreased in *Nfat1*^−/−^ mice, which indicated that *Nfat1* deletion inhibited the excessive activation of microglia and neuroinflammation caused by HA-TBI (Figure 7, right). Interestingly, L-serine treatment did not further decrease the number of activated microglia in the *Nfat1*^−/−^ group (Figures 7A,B). Moreover, *Nfat1* deletion significantly reduced the number of CD16^+^ cells and the ratio of CD16^+^/Iba-1^+^, playing a similar role as L-serine *in vivo* and *in vitro* (Figures 7A,C, *p* < 0.01). Moreover, L-serine treatment did not further decrease the ratio of CD16^+^/Iba-1^+^ cells after the deletion of *Nfat1* (*p* > 0.05). *Nfat1* deletion significantly promoted the expression of CD206^+^ cells and increased the ratio of CD206^+^/Iba-1^+^ cells (Figures 7B,D, *p* < 0.01), but adding L-serine did not increase the ratio of CD206^+^/Iba-1^+^ cells (*p* > 0.05). These data indicate a relevant relationship between NFAT1 and L-serine in modulating the transition of polarization from M1 to M2 microglia.

## 4. Discussion

TBI at HA represents a unique region in the field of medicine and has attracted increasing attention. At present, different altitude areas have been classified internationally, including high altitude areas (3,000–5,000 feet), very high altitude areas (5,000–8,000 feet), and extremely high altitude areas (more than 8,000 feet) (Jain, 2016). These areas are characterized by decreases in temperature and ambient humidity and declining barometric pressure, which causes a decrease in the partial pressure of oxygen. Exposure to low pressure and hypoxia will also lead to physiological reactions to various factors, including altitude reaction, sleep apnea, cognitive dysfunction, pulmonary edema and brain edema (Imray et al., 2010; Jin, 2017). Acute altitude sickness mainly occurs due to oxidative stress caused by hypoxia and low pressure (Dosek et al., 2007). On this basis, the activation of microglia in brain tissue, the proliferation of reactive astrocytes, the release of inflammatory factors and the destruction of the blood–brain barrier caused by TBI will make the injury more serious and the morbidity higher. It is of interest to evaluate the effects of different HAs on TBI and the effective agents to treat it.

In this study, we established an HA-TBI model in mice and compared the neurobehavioral function and histomorphology of acute TBI at different altitudes with those of TBI mice at sea level. We found that compared with those in the TBI group, the neurological functions and histological lesions in the 6,000 m + TBI group and 8,000 m + TBI group were significantly worse. With increasing altitude, the neurological function of mice decreased, the degree of neuronal necrosis and mortality in mice increased, and the damage of cerebral gray matter and white matter became more significant. In the HA-TBI groups with an altitude of 4,000 m and 6,000 m, no mice died within 7 days after TBI, but in the HA-TBI group with an altitude of 8,000 m, the mortality rate was as high as 33%. Based on the above results, we chose a simulated HA environment at an altitude of 6,000 m to study the pharmacology and mechanism of L-serine in the following experiments.

The basic pathological changes after TBI include primary injury and secondary injury (Simon et al., 2017). Neuroinflammation plays an important role in secondary injury following TBI, which is mainly mediated by microglia and astrocytes (Hammad et al., 2018; Pišlar et al., 2021). Microglia are crucial effector cells related to the immune response in the central nervous system (CNS) and play important roles in the inflammatory response (Corrigan et al., 2016). Astrocytes are the most abundant cell type in the CNS, accounting for approximately 30% of the brain volume. However, in CNS diseases, microglia are usually activated earlier than astrocytes (Wang Y. et al., 2022). By generating molecular signals, activated microglia may act as “pioneers” and trigger reactive astrocytes, while astrocytes tend to be less responsive to injury in the absence of microglia (Chen et al., 2019). Inflammatory stimulation causes damage to neurons and oligodendrocytes around the initial injury area, further accelerating the damage to the sensorimotor cortex and white matter, which is related to deficits in sensorimotor function (Wang et al., 2013; Song et al., 2022). There is growing evidence that L-serine regulates the release of several cytokines to restore cognitive function, inhibit inflammation, promote remyelination, and play other neuroprotective roles against neurological injury (Ye et al., 2021). Here, we further found that L-serine could significantly improve the sensorimotor function of mice, reverse the increase in brain lesion volume, promote the repair of cerebral gray matter and white matter, and inhibit the activation of microglia after HA-TBI.

Microglia, as immune cells of the CNS, have different shapes in different immune microenvironments (Umpierre and Wu, 2021). During brain inflammatory reactions, microglia/macrophages have multiple phenotypes, including two polarizing phenotypes, M1 (proinflammatory) and M2 (anti-inflammatory) (Jin et al., 2012). Excessive inflammatory reactions aggravate secondary injury and hinder the repair of nerve function. Moreover, some studies have found that the increase in the M1 type was directly proportional to the degree of damage to oligodendrocytes. However, the M2 phenotype promotes the release of neurotrophic factors that promote remyelination (Chen et al., 2019). We previously found that microglia/macrophages respond to TBI with a transient M2 phenotype, followed by a shift to M1, and that the number of M1 cells is strongly correlated with the severity of TBI (Wang et al., 2013, 2015). However, studies on the regulation of microglial polarization after HA-TBI have seldom been reported. L-serine may have the capability to stimulate NSCs to differentiate into oligodendrocytes and may develop its function in the regulation of the two polarized phenotypes (Ye et al., 2021). Here, we demonstrated that L-serine significantly reduced the activation of microglia after HA-TBI and differentiated microglia/macrophages into the beneficial M2 phenotype, thus playing an anti-inflammatory role.

As *Nfat1* participates in the regulation of early inflammatory genes in microglia and in the phenotype of microglia (Nagamoto-Combs and Combs, 2010), we further investigated the role of NFAT1 in the regulation of the inflammatory response after HA-TBI and whether L-serine promotes the repair of neurological function through the NFAT1 pathway. We found that L-serine significantly inhibited the expression of NFAT1 and reduced microglial activation after HA-TBI. Then, in *Nfat1* knockout mice, we observed a neuroprotective effect similar to the effect of L-serine on WT mice. However, after administration of L-serine to *Nfat1* knockout mice, we did not observe any extra neuroprotective effects, suggesting that L-serine may regulate the polarization of microglia by mediating the expression of NFAT1, thus inhibiting the release of inflammatory factors and alleviating secondary injury after HA-TBI. Microglia have low spontaneous calcium activity under normal conditions, while inflammation and injury increase microglial calcium signalling (Brawek and Garaschuk, 2017; Jiang et al., 2022). A wide variety of extracellular molecules, purine molecules, and immune mediators have been identified to induce calcium transients in microglia (Umpierre and Wu, 2021; Jiang et al., 2022). Data support that *Nfat1* deletion affects the expression of various genes that are mainly associated with immune response-related biological processes, including genes responsible for microglial proliferation (*Csf1r*, *Cx3cr1*, and *P2ry12*) and genes associated with a reactive state of microglia (*Itgam* and *Aif1*) (Jiang et al., 2022). Here, our data demonstrated that *Nfat1* deletion affected the activation degree and number of microglia and inhibited the shift from M2 to M1 microglia, thus alleviating the secondary inflammation caused by HA-TBI.

## 5. Conclusion

**Figure 8:**
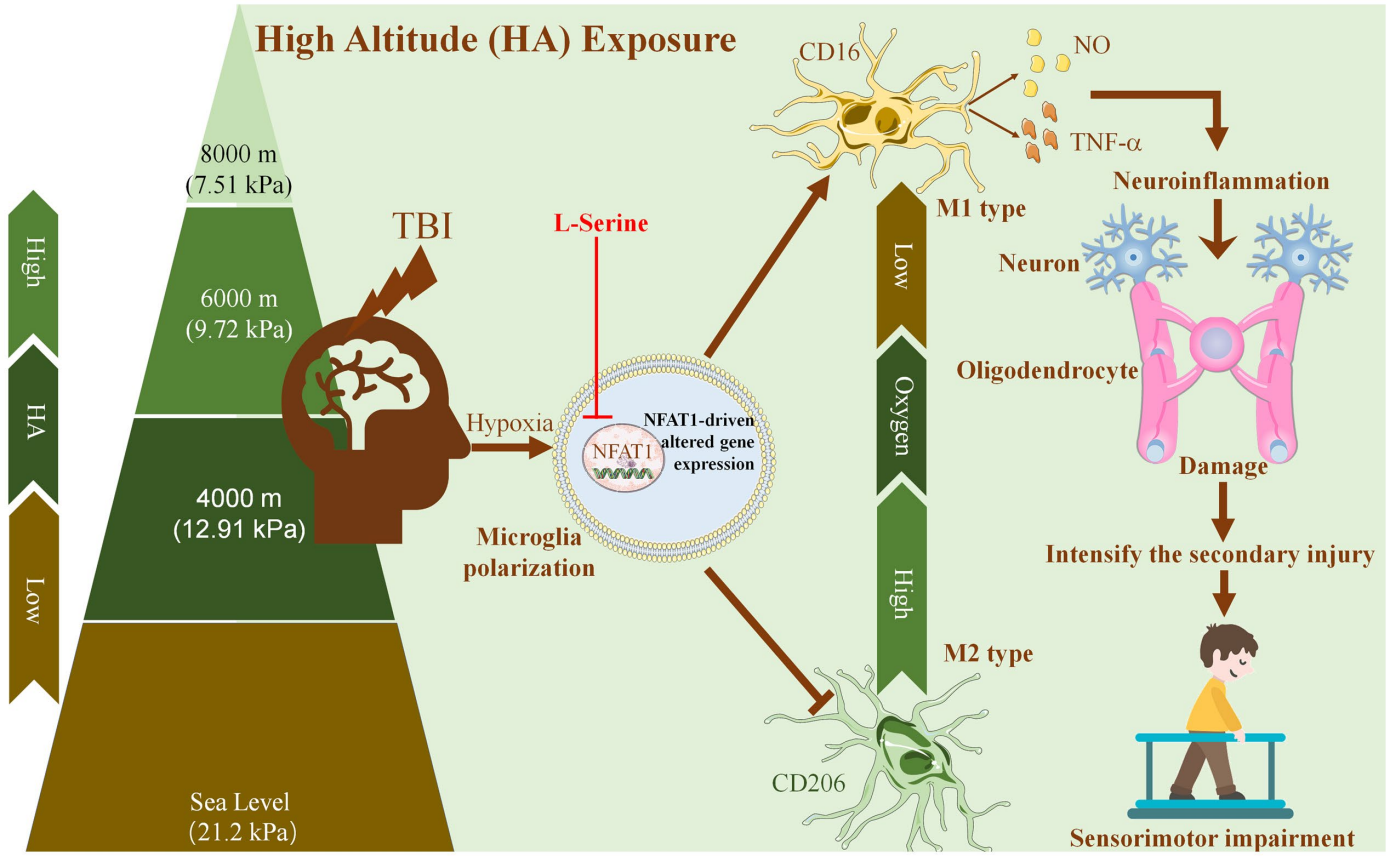
Proposed mechanism underlying the neuroprotective effects of L-serine against traumatic brain injury at high altitude. With increasing altitude, the oxygen partial pressure in the air decreases gradually. HA enhances the activation of microglia and promotes microglial polarization toward the proinflammatory M1 phenotype. HA exposure aggravates neuroinflammation after TBI, which causes injury to neurons and oligodendrocytes around the initial injury area, further aggravates secondary injury to the sensorimotor cortex and white matter, and leads to the deterioration of sensorimotor function. As an endogenous amino acid, L-serine may promote the polarization of microglia to the M2 phenotype and inhibit neuroinflammation by downregulating the transcription factor NFAT1, thus playing a neuroprotective role after HA-TBI and improving sensorimotor function.

In conclusion, (Figure 8), the present study suggests that HA aggravates neuroinflammation in TBI, which causes damage to neurons and oligodendrocytes around the initial injury area and further accelerates secondary injury to the sensorimotor cortex and white matter. Moreover, the severity of HA-TBI damage is altitude-dependent, which is related to deficits in sensorimotor function. As an endogenous amino acid, L-serine offered significant neuroprotection of both gray matter and white matter after HA-TBI. Mechanistically, L-serine may promote microglial polarization toward the M2 phenotype and suppress inflammation *via* the downregulation of the transcription factor NFAT1. Thus, L-serine may be a neuroprotective agent against HA-TBI, and suppression of NFAT1 in microglia is a potential future treatment for neuroinflammation. This study provides a new target with potential application value for the prevention and intervention of TBI in HA environments.

## Data availability statement

The raw data supporting the conclusions of this article will be made available by the authors, without undue reservation.

## Ethics statement

The animal study was reviewed and approved by the Animal Ethics Committees of Nantong University.

## Author contributions

GW and MH conceived, organized, and supervised the work. JL, SP, LY, and YS performed the experiments. QZ and HW contributed to the analysis of data. JL, QL, and GW prepared, wrote and revised the manuscript. All authors contributed to the article and approved the submitted version.

## Funding

This work was supported by the Natural Science Foundation of China (grants 82171190 and 81873924), Key Scientific Research Projects of Colleges and Universities in Henan Province in 2021 (grant 22B310001), China Postdoctoral Science Foundation (2020 M673649) and Nantong Municipal Science and Technology Project (MS12021020 and MS22021010).

## Conflict of interest

The authors declare that the research was conducted in the absence of any commercial or financial relationships that could be construed as a potential conflict of interest.

## Publisher’s note

All claims expressed in this article are solely those of the authors and do not necessarily represent those of their affiliated organizations, or those of the publisher, the editors and the reviewers. Any product that may be evaluated in this article, or claim that may be made by its manufacturer, is not guaranteed or endorsed by the publisher.
